# Fibrinolysis in a lipid environment: modulation through release of free fatty acids

**DOI:** 10.1111/j.1538-7836.2007.02556.x

**Published:** 2007-06-01

**Authors:** G RÁBAI, B VÁRADI, C LONGSTAFF, P SÓTONYI, V KRISTÓF, F TIMÁR, R MACHOVICH, K KOLEV

**Affiliations:** * Department of Medical Biochemistry, Semmelweis University Budapest, Hungary; † Division of Biotherapeutics, National Institute for Biological Standards and Control Potters Bar, Hertfordshire, UK; ‡ Department of Cardiovascular Surgery, Semmelweis University Budapest; § 1st Department of Pathology, Semmelweis University Budapest, Hungary

**Keywords:** fibrinolysis, oleic acid, plasmin, reteplase, tissue-type plasminogen activator

## Abstract

*Background:* Thrombolysis is conventionally regarded as dissolution of the fibrin matrix of thrombi by plasmin, but the structure of clots *in vivo* includes additional constituents (proteins, phospholipids) that modulate their solubilization. *Objective:* We examined the presence of free fatty acids in thrombi and their effects on distinct stages of fibrinolysis (plasminogen activation, plasmin activity). *Methods and Results:* Using the fluorescent probe acrylodated intestinal fatty acid-binding protein, variable quantities (up to millimolar concentrations) of free fatty acids were demonstrated in surgically removed human thrombi. Oleic acid at relevant concentrations reversibly inhibits more than 90% of the amidolytic activity of plasmin on a synthetic substrate (Spectrozyme PL), but only partially inhibits its fibrinolytic activity measured using turbidimetry. Chromogenic assays detecting the generated plasmin activity show that plasminogen activation by tissue-type plasminogen activator (t-PA) is completely blocked by oleic acid in the fluid phase, but is accelerated on a fibrin matrix. A recombinant derivative of t-PA (reteplase) develops higher fibrin specificity in the presence of oleic acid, because both the inhibition of plasminogen activation in free solution and its enhancement on fibrin template are stronger than with wild-type t-PA. *Conclusion:* Through the stimulation of plasminogen activation on a fibrin template and the inhibition of plasminogen activators and plasmin in the fluid phase, free fatty acids confine the action of fibrinolytic proteases to the site of clotting, where they partially oppose the thrombolytic barrier function of phospholipids.

## Introduction

When thrombi are formed within blood vessels, cells (platelets and leukocytes) infiltrate the fibrin matrix and later profoundly affect proteolytic dissolution by plasmin (reviewed in [1,2]). The recently described thrombolytic barrier function of phospholipids [3] is based on diffusion limitations and intermolecular interactions with fibrinolytic enzymes. In a phospholipid environment, both the conversion of plasminogen to plasmin by plasminogen activators [e.g. tissue-type plaminogen activator (t-PA)] and the proteolytic action of plasmin are significantly retarded. Although there are only traces of free fatty acids in resting platelets [4], as part of their activation mechanism phospholipase A_2_ (PLA_2_) stored in secretory granules is released and activated [5–7]. Thus, PLA_2_ hydrolyzes the ester bond at the *sn*-2 position of the phospholipids, releasing a free fatty acid and lysophospholipid. Considering the millimolar concentration of phospholipids in arterial thrombi [3], PLA_2_ from entrapped cellular elements (platelets and leukocytes) could, hypothetically, release large quantities of free fatty acids (mainly oleic acid, because it occupies more than 54% of the *sn*-2 ester bonds in platelet phospholipids [4]).

The data on the impact of free fatty acids on the fibrinolytic system are scarce and controversial. The amidolytic activity of plasmin on small synthetic substrates is reported to be stimulated by oleic acid [8,9], whereas plasmin digestion of macromolecules (fibrin, prostromelysin-1) is inhibited [9,10]. The modulation of the plasmin activity is probably mediated by the binding of oleic acid to the kringle-5 domain of the enzyme [9]. Among the examined fatty acids with varying numbers of carbon atoms and double bonds, oleic acid proved to be the most potent in its effect on the action of plasmin [9,10]. The influence of fatty acids on plasminogen activation is even less well characterized. Only one plasminogen activator (urokinase) has been evaluated in this respect, and stimulation of its activity by oleic acid has been reported [9,10]. No data on the modulation of other activators and the inhibitors of fibrinolysis are available.

The thrombolytic agents currently approved for treatment of myocardial infarction and ischemic stroke are all plasminogen activators with more or less frequent hemorrhagic side-effects, which led to a search for new fibrin-specific plasminogen activators. However, administration of fibrin-binding thrombolytics (t-PA) did not overcome the bleeding complications [11]. Reteplase is a recombinant variant of t-PA, which consists of the kringle-2 and the protease domains of the wild-type activator (reviewed in [12]). As expected from the lack of the finger domain, reteplase has lower affinity for fibrin, but clinical trials have shown that it produces faster reperfusion than t-PA, even at lower doses, and without any increase in the rate of bleeding side-effects, despite a moderate decrease in fibrinogen levels [13]. This apparent discrepancy between the clinical profile and the lack of fibrin selectivity of reteplase can be partially explained by its slower clearance in circulation, but, as our present study shows, evaluation of the activator in models that approach the complex composition of thrombi provides better understanding of its *in vivo* thrombolytic properties.

## Methods

### Preparation of LUVs

Dipalmitoyl-phosphatidylcholine (PC), dipalmitoyl-phosphatidylserine (PS), palmitoyl-oleoyl-phosphatidylcholine (poPC) and lysophosphatidylcholine (1-palmitoyl) (lysoPC) (Sigma-Aldrich Kft., Budapest, Hungary) were weighed, mixed at the desired mass ratio, and dissolved in chloroform/methanol (95:5 volume ratio). Following evaporation of the solvent, the phospholipids were suspended with brief sonication, and large unilamellar phospholipid vesicles (LUVs) were prepared by extrusion through a 50-nm-diameter polycarbonate filter in a Liposofast mini-extruder (Avestin Inc., Ottawa, Canada) [16]. The concentration of phospholipids in the LUV suspension was determined with the fluorescent probe 1,6-diphenyl-1,3,5-hexatriene [17]. The LUVs designated as poPCPS contained poPC, PC and PS mixed at 2:3:5 weight ratio.

### Turbidimetric fibrinolytic assay

This was carried out as previously described [15]. Briefly, 100 μL of 6 μmol L^−1^ fibrinogen (Calbiochem, LaJolla, CA, USA) containing 0.25 μmol L^−1^ plasminogen (isolated from human plasma [18]), 1 mg mL^−1^ LUVs (poPCPS) and 0.5 mg mL^−1^ PLA_2_ (Sigma-Aldrich Kft., Budapest, Hungary) was clotted with 0.01 μmol L^−1^ thrombin in microplate wells at 37 °C for 45 min. The dissolution of the clots was induced by 100 μL of t-PA (Boehringer Ingelheim, Ingelheim, Germany) applied on the surface of the clot at concentrations in the range 10–120 nmol L^−1^, and 60 μL of mineral oil was layered over the solution to prevent evaporation. The course of clot formation and dissolution was monitored by measuring the light absorbance at 340 nm at 37 °C with a Zenyth 200rt microplate spectrophotometer (Anthos Labtec Instruments GmbH, Salzburg, Austria). The lysis time (*t*_1/2_), defined as the time needed to reduce the turbidity of the clot to a half-maximal value, was used as a quantitative parameter of fibrinolytic activity. In certain experiments, α_2_-plasmin inhibitor (α_2_PI; Sigma-Aldrich Kft., Budapest, Hungary) was added to fibrinogen prior to clotting.

### Plasminogen activation assays

Plasminogen activation in homogeneous solution was evaluated as previously described [14]. Briefly, 3 μmol L^−1^ plasminogen containing fatty acids at various concentrations (a 14 mmol L^−1^ stock solution of fatty acids in ethanol was further diluted in 10 mmol L^−1^ HEPES buffer (pH 7.4) containing 150 mmol L^−1^ NaCl) was mixed with 70 nmol L^−1^ t-PA, samples were taken at intervals, and the amidolytic activity of the generated plasmin was measured on 0.1 mmol L^−1^ Spectrozyme-PL (*H*-d-norleucyl-hexahydrotyrosyl-lysine-*p*-nitroanilide; American Diagnostica, Hartford, CT, USA). Ethanol at concentrations equivalent to the amount included in the reaction mixtures as a solvent of fatty acids did not affect this assay, in agreement with an earlier report [8]. Plasminogen activation in the presence of fibrin was measured on the surface of clear fibrin clots (*A*_405_ < 0.1) [19] prepared in standard 96-well microtiter plates from 20 μL of 0.2 μmol L^−1^ thrombin and 60 μL of 4.4 μmol L^−1^ fibrinogen in 10 mmol L^−1^ HEPES (pH 7.4) buffer containing 200 mmol L^−1^ NaCl and 0.1 μmol L^−1^ plasminogen. After 30 min of incubation at 37 °C, 60 μL of plasminogen activator (t-PA or reteplase from Centocor, Malvern, PA, USA) in 10 mmol L^−1^ HEPES (pH 7.4) buffer containing 150 mmol L^−1^ NaCl and 0.6 mmol L^−1^ Spectrozyme-PL or S-2251 (*H*-d-valyl-l-leucyl-l-lysine-*p*-nitroanilide; Chromogenix SpA, Milan, Italy) was layered on the clot surface, followed by 60 μL of mineral oil. The light absorbance at 405 nm (*A*_405_) indicating the release of *p*-nitroaniline by the generated plasmin was continuously recorded with a Zenyth 200rt microplate spectrophotometer at 37 °C. The measured *A*_405_ values were plotted vs. time squared, yielding a linear relationship with a slope directly proportional to the rate of plasminogen activation. If, at time *t*_0_ = 0, there was no plasmin in the reaction system, then according to [20], Δ*A*_405_ = 0.5*εk*_1_*k*_2_[PA]*t*^2^, where *ε* is the extinction coefficient for *p*-nitroaniline (3.82 mmol^−1^ L determined for the volume of our assay system), *k*_1_*=* 13.5 s^−1^ is the turnover number of plasmin on Spectrozyme PL [21], and *k*_2_ and [PA] are the apparent reaction rate constant for plasminogen activation in this assay and the concentration of the plasminogen activator, respectively.

### Removal of fatty acids from serum albumin

Fatty acids were removed from the commercial bovine serum albumin (BSA; Sigma-Aldrich Kft., Budapest, Hungary) by charcoal treatment at pH 2.5 according to a published procedure [22]. Protein concentration was determined from the absorbance at 280 nm using an extinction coefficient of 0.67 for the 1 g L^−1^ albumin solution.

### Free fatty acid release from phospholipids

The release of free fatty acids from LUVs by PLA_2_ was measured with the fluorescent probe acrylodated intestinal fatty acid-binding protein (ADIFAB) (Molecular Probes, Leiden, the Netherlands) [23]. LUVs (1 g L^−1^ phospholipid concentration) were incubated with 0.5 mg mL^−1^ PLA_2_ in 10 mmol L^−1^ HEPES (pH 7.4) buffer containing 150 mmol L^−1^ NaCl and 3 mmol L^−1^ CaCl_2_ in the presence of 0.2 μmol L^−1^ ADIFAB at 37 °C in a 1-mL fluorimetric cuvette. The emission ratio at 505 and 432 nm (excitation 386 nm) was monitored continuously for 30 min with a Photon Technology International (PTI; Lawrenceville, NJ, USA) Deltascan fluorescence spectrophotometer. The free fatty acid concentration was determined from a calibration curve generated for known concentrations of sodium oleate in the presence of 1 g L^−1^ LUVs.

### Detection of free fatty acids in thrombi

Thrombi were surgically removed from the femoral artery, aortoiliac aneurysm, femoropopliteal graft or saphenous vein of four patients with hyperlipidemia and generalized atherosclerosis. Patients gave informed consent, and permission was obtained from the local ethics committee. After the thrombectomy, the samples were frozen immediately at – 70 °C and stored until examination. Cryosections (5 μm × 1.5 cm × 1.5 cm) of thrombi were prepared without fixation, and immersed in 100 μL of 2 μmol L^−1^ ADIFAB on glass plates for 10 min. Thereafter, the spatial pattern of fluorescence at 460 nm (excitation 390 nm) was measured with a scanning fluorescence spectrophotometer (Fluoroskan Ascent FL, Labsystems Oy, Helsinki, Finland).

## Results

**Fig. 1:**
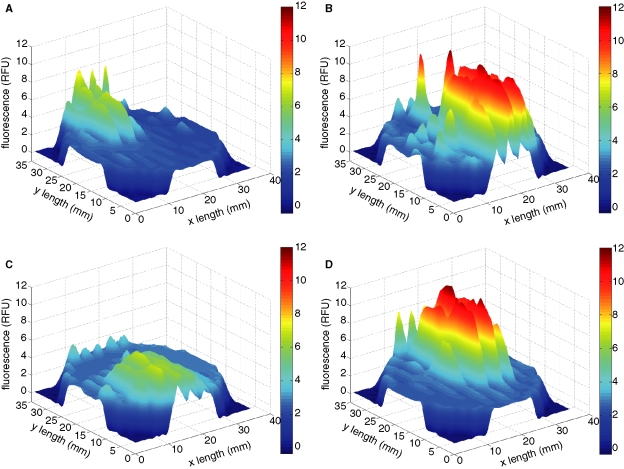
Presence of free fatty acids in thrombi. Cryosections of thrombi were prepared and exposed to 100 μL of 2 μmol L^−1^ acrylodated intestinal fatty acid-binding protein (ADIFAB) for 10 min. The fluorescence at 460 nm (excitation 390 nm) was measured in 400 points of the section plane area with dimensions *x* and *y*, and presented in relative fluorescence units (RFU). (A) and (B) are representative scans of thrombi with high and low fatty acid content, respectively. (C) shows the fluorescence of 100 μL of 2 μmol L^−1^ ADIFAB containing 20 μmol L^−1^ sodium oleate, and (D) shows the fluorescence of 100 μL of 2 μmol L^−1^ ADIFAB in the absence of fatty acid.

Surgically removed human thrombi were examined for the presence of free fatty acids and estimation of their concentration. The probe ADIFAB indicated highly variable amounts of free fatty acids in the thrombectomy samples. In five of eight thrombi examined by us, the free fatty acids present in the thrombus sections completely quenched the fluorescence of the applied ADIFAB (Fig. 1A), which is equivalent to 20 μmol L^−1^ sodium oleate in the same volume of ADIFAB (Fig. 1C). The original fluorescence of ADIFAB (Fig. 1D), however, was not changed in three of the examined thrombi (Fig. 1B). Thus, the amount of free fatty acids in thrombi spans a range from undetectable levels up to millimolar concentrations (fatty acids from 1 μL of thrombus saturate 0.2 nmol L ADIFAB).

The effects of fatty acids on fibrinolysis were analyzed with *in vitro* assay systems. We modeled the overall course of thrombolysis induced by t-PA under the changing conditions of ongoing hydrolysis of phospholipids with fibrin–phospholipid clots containing PLA_2_ (at a concentration releasing 1.97 μmol L^−1^ min^−1^ fatty acid from LUVs). In order to identify the effects of the phospholipid hydrolytic products, which are not related simply to the elimination of the phospholipids, we assembled fibrin clots with a poPCPS mixture, the melting temperature of which is 33 °C [24]. This phospholipid only mildly affected the course of fibrinolysis at 37 °C, in agreement with our previous findings that an ordered gel phase is required for the inhibitory effects of phospholipids on clot dissolution [3]. However, when the concentration of free fatty acid released by PLA_2_ increased to over 0.24 mmol L^−1^ (measured with ADIFAB), the dissolution process was significantly accelerated. Because LUVs containing lysophospholipid (the other product of the PLA_2_ action on phospholipids) at the same concentration did not affect the clot dissolution, we restricted our further experiments to the effects of fatty acids.

**Fig. 2:**
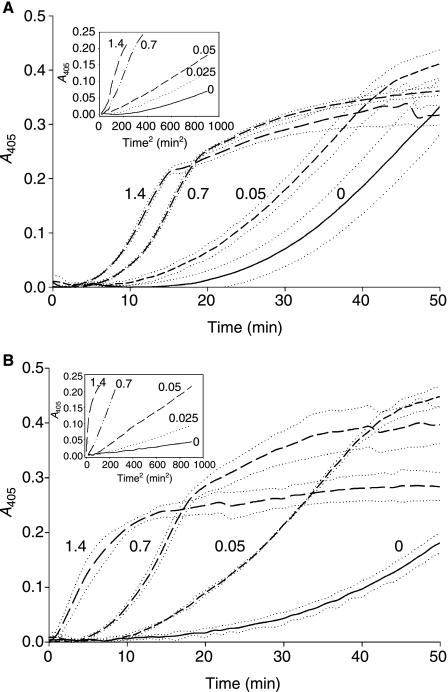
Effect of oleic acid on plasminogen activation in the presence of fibrin. Clear fibrin clots containing plasminogen were prepared as described in Methods, and 0.7 nmol L^−1^ tissue-type plasminogen activator (t-PA) (A) or reteplase (B) was added together with the plasmin substrate Spectrozyme PL and varying concentrations of oleic acid, indicated by the numbers (in mmol L^−1^) next to the lines. The continuously monitored *A*_405_ is presented as mean and SD (dotted lines) of five measurements. Insets: secondary plots of *A*_405_ vs. time squared; the slopes are linearly proportional to the rate of plasmin generation.

Using an assay specific for plasminogen activation on a fibrin surface [19], significant acceleration of plasmin generation was measured in the presence of oleic acid with the fibrin-binding activators t-PA and reteplase (Fig. 2), but not with the non-fibrin-binding activator urokinase (not shown). The activation with reteplase was more sensitive to the effect of the oleic acid than that with t-PA; identical fatty acid concentrations caused greater stimulation of the reaction rate with reteplase (Fig. 2, insets; Table 1). Because Spectrozyme-PL is known to affect plasminogen activation under certain conditions, whereas S-2251 does not [25], the experiments shown in Fig. 2 were repeated with this alternative plasmin substrate. At concentrations of oleic acid up to 0.5 mmol L^−1^, the assay with S-2251 showed the same pattern of acceleration (data not shown), indicating that the observed stimulation does not depend on the type of detection substrate. At higher oleic acid concentrations, however, the absorbance progress curves reached plateaus at much lower values than in the Spectrozyme-PL system, resulting in a loss of sensitivity, which can be attributed to the different *K*_m_ values of the two substrates for plasmin (266 μmol L^−1^ for S-2252 [19] vs. 10 μmol L^−1^ for Spectrozyme-PL [14]).

**Table 1:** Relative efficiency of plasminogen activation in the presence of oleic acid on fibrin surface

Oleic acid (mmol L^−1^)	t-PA [mean (SD)]	Reteplase [mean (SD)]
0	1.00 (0.22)	1.00 (0.17)
0.025	1.29 (0.21)	1.78 (0.25)
0.05	1.81 (0.29)	4.18 (0.54)
0.7	7.37 (1.24)	14.54 (2.09)
1.4	10.19 (1.93)	35.78 (4.66)

The slopes of the linear plots in Fig. 2 (insets) reflect the rate of plasmin generation in accordance with the equation given in Methods. The ratios of these slopes in the presence of oleic acid and in its absence are calculated for the respective activator, and their values are presented as mean and SD (in parentheses) of five measurements determined with a bootstrap procedure for a ratio of two measured variables. The ratio of the baseline rates (in the absence of oleic acid) of plasmin formation by equimolar concentrations of the two activators (reteplase/tPA) is 0.54 (0.11).

t-PA, tissue-type plasminogen activator.

**Fig. 3:**
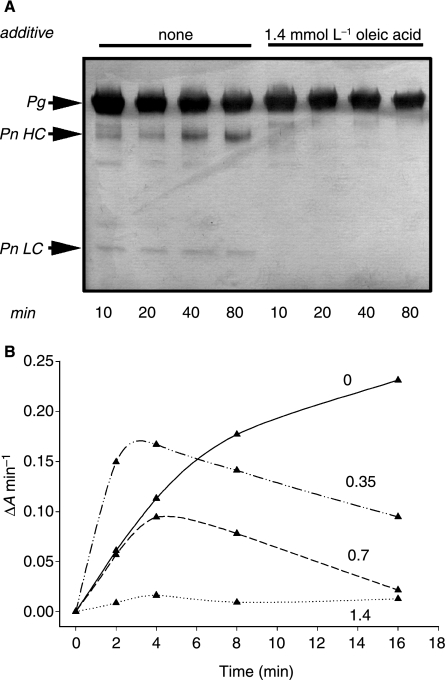
Effect of oleic acid on plasminogen activation in solution. (A) The activation mixture contained 1 μmol L^−1^ plasminogen and 7 nmol L^−1^ tissue-type plasminogen activator (t-PA). Samples taken at the indicated time were treated with 100 mmol L^−1^ Tris–HCl (pH 7.0) buffer containing 100 mmol L^−1^ NaCl, 2% sodium dodecylsulfate, and 1%β-mercaptoethanol, and following electrophoresis on 10–15% polyacrylamide gel, the protein bands were visualized with silver staining. Pg, plasminogen; Pn HC and Pn LC, plasmin heavy and light chains, respectively. (B) The activation mixture contained 3 μmol L^−1^ plasminogen, 100 μg mL^−1^ cyanogen bromide human fibrinogen fragment, and 70 nmol L^−1^ t-PA. Samples were taken at the times indicated by symbols, and the amidolytic activity of the generated plasmin was measured on 0.1 mmol L^−1^ Spectrozyme-PL. The concentration of oleic acid in the activation stage of the assay is indicated by the numbers next to the lines in mmol L^−1^. The mean values of two independent measurements are presented.

The template function of fibrin seems to be essential for the stimulatory effect of oleic acid, because no activation was seen in the absence of template (Fig. 3A), and only a transient plasmin activity could be detected in the presence of a soluble template, cyanogen bromide human fibrinogen fragment (FgDP) (Fig. 3B). The loss of plasmin activity in the experimental setting of Fig. 3B, as well as in the later stages of the fibrin-dependent plasminogen activation assay (Fig. 2), was a surprising result in the light of earlier reports on stimulation of the plasmin amidolytic activity on small substrates by oleic acid [8,9], and prompted evaluation of the activator and plasmin activities in fibrin-free systems.

**Fig. 4:**
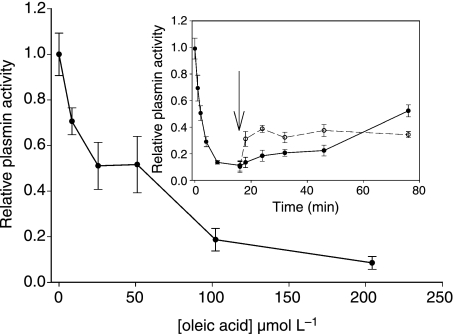
Inhibition of the amidolytic activity of plasmin by oleic acid. Plasmin at 25 nmol L^−1^ was incubated with oleic acid for 15 min at 37 °C in 10 mmol L^−1^ HEPES buffer (pH 7.4) containing 150 mmol L^−1^ NaCl. Then, 180 μL of this mixture was added to 20 μL of 1 mmol L^−1^ Spectrozyme PL, and the *A*_405_ was measured continuously for 1 min. The ratio of Δ*A* min^−1^ in the presence and absence of fatty acid is presented as relative plasmin activity (mean and SD of five measurements). Inset: time dependence of the inhibition of plasmin by oleic acid. The same measurement was performed with plasmin incubated with 100 μmol L^−1^ oleic acid for various time intervals. At the times indicated by arrows, fatty acid-free bovine serum albumin was added to the plasmin solutions (both the oleic acid-free reference and the oleic acid-treated one) at 140 μmol L^−1^ final concentration (solid line), or the reaction mixture was diluted 5-fold with buffer (dashed line), and plasmin activity was monitored further.

**Fig. 5:**
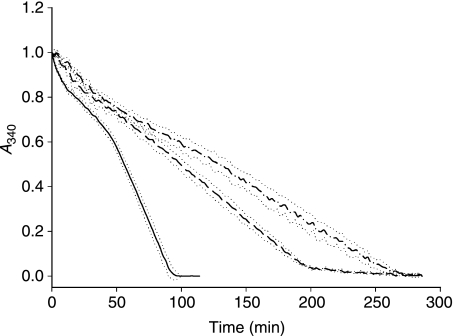
Inhibition of the fibrinolytic activity of plasmin by oleic acid. Fibrin clots were prepared from 6 μmol L^−1^ fibrinogen as described in Methods, omitting the plasminogen. After 30 min, 60 μL of 10 μmol L^−1^ plasmin was added to the surface of the clot, followed by 60 μL of mineral oil. The plasmin solution contained no additive (solid line), 0.35 mmol L^−1^ oleic acid (dashed line), or 1.4 mmol L^−1^ oleic acid (dashed-and-dotted line). Means and SDs (dotted lines) of five measurements are presented.

A spurious stimulation of the amidolytic activity of plasmin by oleic acid was seen when the action of 1 nmol L^−1^ plasmin on 0.1 mmol L^−1^ Spectrozyme PL was monitored for a period of 30 min in an assay system free of additional proteins and detergents. This effect, however, is related to adsorption of the enzyme to the plasticware, which is prevented by the detergent effect of the fatty acid. When shorter incubation with a higher concentration of plasmin (25 nmol L^−1^) was used in the amidolytic assay, the magnitude of this interference was negligible, and a definite inhibition of the plasmin activity was observed (Fig. 4). Plasmin inhibition could be reversed slowly by BSA, which binds the free fatty acids, or by dilution of the plasmin–oleic acid mixture (Fig. 4, inset). Similar (but weaker) effects on the amidolytic activity of plasmin are produced by arachidonic acid (data not shown). The fibrinolytic activity of plasmin, however, was not affected to the same degree as its amidolytic activity (Fig. 5). The presence of oleic acid in the fibrin clot did not affect the lysis rate with plasmin applied to its surface without oleic acid (data not shown).

**Fig. 6:**
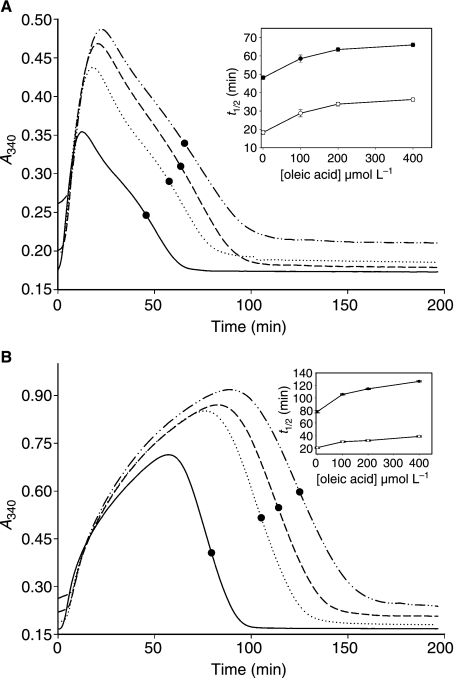
Effects of oleic acid on plasminogen activation and fibrin(ogen) degradation. One hundred microliters of 6 μmol L^−1^ fibrinogen containing 10 nmol L^−1^ plasminogen and various concentrations of oleic acid (0, continuous line; 100 μmol L^−1^, dotted line; 200 μmol L^−1^, dashed line; 400 μmol L^−1^, dashed-and-dotted line) was added to 5 μL of 0.2 μmol L^−1^ thrombin and 5 μL of 100 nmol L^−1^ tissue-type plasminogen activator (A) or reteplase (B) in microplate wells, and after layering 60 μL of mineral oil on the surface, the absorbance at 340 nm was recorded continuously at 37 °C. Mean values of four measurements are presented. Symbols indicate lysis time (*t*_1/2_). Insets: *t*_1/2_ values from the start of the reactions (filled symbols) and after maximal *A*_340_ (open symbols) from the same experiments, mean ± SD.

The combination of plasminogen activation in fluid phase and on a fibrin template in a single assay provided further evidence for the role of fibrin in the modulating effects of oleic acid on plasminogen activation and plasmin activity described above. When fibrinogen clotting and plasminogen activation were initiated simultaneously (Fig. 6), during the ascending phase of the turbidity curves plasminogen activation occurred in a fluid fibrinogen environment, where plasmin is susceptible to the action of oleic acid. Thus, at increasing oleic acid concentrations, more fibrinogen was spared from plasmin digestion, resulting in higher values of absorbance (oleic acid on its own does not affect the clot turbidity in the absence of plasmin formation; data not shown). During the descending phase of the curves, when most of the fibrinogen had already been converted to fibrin, the fibrinolytic rate was hardly affected by oleic acid (lysis time after maximal *A*_340_ in Fig. 6, insets). The effect of oleic acid depended on the type of activator; although less efficient in the fluid phase [26], reteplase showed fibrinolytic efficiency similar to that of t-PA in the fibrin-dependent stage of the assay (lysis time after maximal absorbance in Fig. 6A,B). In agreement with earlier reports [9,10], oleic acid stimulated plasminogen activation by urokinase in this assay (data not shown). If 3 μmol L^−1^ plasminogen and 30 nmol L^−1^ t-PA or reteplase were added together with oleic acid (in the range 0.01–0.4 mmol L^−1^) to the surface of plasminogen-free fibrin clots, a concentration-dependent prolongation of the lysis time was detected (from 74.0 ± 4.9 min in the absence of oleic acid to 87.6 ± 2.6 min at the highest oleic acid concentration with t-PA, and from 81.5 ± 5.0 min to 92.5 ± 3.0 min for reteplase).

**Fig. 7:**
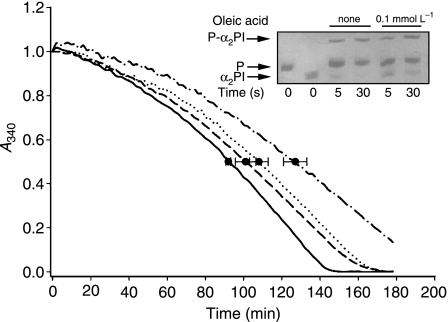
Effect of oleic acid on the plasmin inactivation by α_2_-plasmin inhibitor (α_2_PI). Fibrin clots containing plasminogen were prepared as described in Methods, and dissolution was initiated with 10 nmol L^−1^ tissue-type plasminogen activator (t-PA) and monitored by measuring absorbance at 340 nm. Some clots (dashed, dashed-and-dotted lines) contained 0.25 μmol L^−1^α_2_PI, whereas some t-PA solutions contained 0.3 mmol L^−1^ oleic acid (dotted, dashed-and-dotted lines). Symbols indicate lysis time (mean ± SD of five measurements). Inset: effect of oleic acid on plasmin–α_2_PI complex formation. Plasmin (P, 0.25 μmol L^−1^) and α_2_PI (0.1 μmol L^−1^) were incubated for the indicated times at 20 °C in the absence and presence of oleic acid. Samples of the reaction mixture were taken at the indicated times (0 indicates sampling before mixing plasmin and α_2_PI) and treated with 100 mmol L^−1^ Tris–HCl (pH 7.0) buffer containing 100 mmol L^−1^ NaCl and 2% sodium dodecylsulfate. Following electrophoresis on 12.5% polyacrylamide gel, the protein bands were visualized with silver staining.

Despite the decrease in the amidolytic and fibrinolytic activity of plasmin caused by oleic acid (Figs 4 and 5), the susceptibility of the protease to its major plasma inhibitor, α_2_PI was not modified significantly in the fluid phase (Fig. 7, inset). As a result, oleic acid and α_2_PI (at the concentration detected in human thrombi [27]) yielded additive inhibition of plasmin in fibrin clots (Fig. 7).

## Discussion

Considering the known phospholipid content and PLA_2_ release from platelets and inflammatory cells in thrombi [3,5–7], the thrombus localization of free fatty acids is not a surprising finding, but their direct demonstration in human thrombi (Fig. 1) is important evidence for the *in vivo* relevance of their effects described by others [8–10] or reported now with respect to thrombolysis. In addition, because of known inhibition of other proteases by oleic acid (leukocyte elastase, gelatinases A and B released by inflammatory cells) [28,29], the identification of free fatty acids in the structure of thrombi at varying (up to millimolar) concentrations supports their *in vivo* role in the modulation of inflammation-related proteolysis in this compartment. Because of the abundance of oleoyl side chains in platelet phospholipids [4] and the reported data for the role of unsaturated long-chain (more than 16 carbon atoms) fatty acids in the modulation of plasmin and other proteases [9,10,28], oleic acid was chosen as a model molecule.

The overall concept that emerges from our results is that oleic acid is a factor that contributes to the localized action of plasmin in fibrin clots; it favors plasmin generation on its surface, and at the same time prevents the dissemination of protease activity in the circulation because of its inhibitory effects in solution. Using an assay that selectively monitors plasminogen activation at the interface of the fluid phase (containing activator) and fibrin (containing plasminogen) [19], significant acceleration of plasmin generation by t-PA can be demonstrated in the presence of oleic acid (Fig. 2A). A tenfold increase in the activation rate by t-PA can be achieved within the range of oleic acid concentrations relevant for thrombi (Table 1). This accelerating effect does not require the fibronectin finger-like, the epidermal growth factor and kringle-1 domains of t-PA. Reteplase, a recombinant variant of t-PA containing the kringle-2 and protease domains, is stimulated more strongly by oleic acid on a fibrin surface (Fig. 2B, Table 1). The facts that kringle-2 is involved primarily in interactions with lysine residues [30], which are continuously exposed when plasmin digests fibrin, and that the effect of oleic acid is seen only in the presence of fibrin suggest that the acceleration of plasmin generation is based on optimized formation of the ternary plasminogen–fibrin–activator complex. The inhibition of plasminogen activation in the fluid phase (Fig. 3A) is reversed on the fibrin surface (Fig. 2). Such an effect can be expected if the modulator (oleic acid) causes the template (fibrin) to have higher affinity for the activator, so that more t-PA or reteplase is mobilized in the reactive interfacial layer, and in addition the bound form of the activator is less susceptible to the inhibitory effect of oleic acid. This interpretation is in line with the lack of an accelerating effect of oleic acid on plasminogen activation by urokinase, which does not require a template. Thus, the presence of oleic acid in thrombi may contribute to the therapeutic advantage of fibrin-specific plasminogen activators. On the other hand, because the oleic acid effects vary between fibrin-dependent activators, its role as a fibrinolytic modulator *in vivo* may explain the lack of strict correlation between fibrin binding and therapeutic effectiveness of thrombolytics, in addition to the role of other known factors, such as different pharmacokinetics of the activators *in vivo*.

In contrast to the situation with urokinase [10], t-PA-catalyzed plasminogen activation is completely blocked by oleic acid in fibrin-free solution (Fig. 3A), which is related to the effect of oleic acid on t-PA (its amidolytic activity on small synthetic substrates is also inhibited; data not shown). CNBr digestion of fibrinogen exposes the binding sites for t-PA and plasminogen that are necessary for its template function in plasminogen activation (reviewed in [31]), and consequently FgDP can at least partially relieve this inhibitory effect (Fig. 3B). However, the already formed plasmin is also inhibited by oleic acid in its amidolytic action in the fluid phase (Fig. 4) and to a lesser extent in its fibrinolytic action (Fig. 5). In the absence of an extensive fibrin matrix, FgDP cannot protect the already formed plasmin against the inhibitory effect of oleic acid, and consequently plasmin is not detected in the late stages of the activation assay with this soluble template. A similar phenomenon is observed in the later stages of the clot-dependent plasminogen activation assay (Fig. 2), when the generated plasmin completely digests the interfacial fibrin layer to soluble products. The loss of enzyme activity cannot be attributed to denaturation as a result of the detergent effects of oleic acid, because the reactivity of plasmin with macromolecular inhibitors is retained. In the presence of oleic acid, the sensitivity of plasmin to its natural inhibitor α_2_PI is preserved not only in free solution (Fig. 7, inset), but even on the fibrin surface (Fig. 7), which on its own is known to protect the protease against α_2_PI [21,32]. Plasmin inhibition is reversible (Fig. 4, inset), but the exact type of inhibition requires further investigation. Probably, plasmin inhibition is based on conformational changes in the protease domain induced by the known binding of oleic acid to kringle-5 [9], whereas the kringle-1-dependent interaction with α_2_PI [33] is not affected.

The effects of oleic acid on plasminogen activation and plasmin activity contribute to a better understanding of the *in vivo* data for fibrinolytic potency and fibrin specificity of reteplase, and emphasize the need to perform enzymological evaluation of thrombolytics in an environment that contains not only fibrin but also other modulators identified in thrombi. Thus, reteplase is reported to have 2-fold to 4-fold lower activator efficiency than the wild-type t-PA and to bind weakly to native fibrin because of the lack of the finger domain [26,34], but as a thrombolytic agent, it produces identical or even improved clinical outcome [13]. Our data demonstrate a 2-fold lower apparent reaction rate constant for plasminogen activation by reteplase as compared to t-PA in plasminogen activation on a fibrin surface (Fig. 2, insets), but this difference is reversed in favor of reteplase in the presence of 1.4 mmol L^−1^ oleic acid (Table 1). When the action of the activators is evaluated in a model system (Fig. 6), which monitors the consequences of plasminogen activation with respect to both fibrinogen and fibrin, oleic acid causes reteplase to have higher fibrin specificity and fibrinogen-sparing properties as compared to t-PA, because its common inhibitory effect on plasmin is combined with more pronounced fibrin-dependent stimulation of plasminogen activation by reteplase. This conclusion is based on the changes in lysis time at increasing concentrations of oleic acid (Fig. 6, insets); because reteplase is less efficient in solution [26], the smaller amounts of plasmin generated by it are easily blocked by oleic acid (compare the ascending phase of the turbidity curves in Fig. 6A,B), whereas there is hardly any difference in the fibrinolytic rate with the two activators when plasminogen is activated predominantly on partially degraded fibrin, to which both activators can bind through their kringle-2 domains (descending phase of the curves in Fig. 6). The experimental setting of Fig. 6 also models the action of plasminogen activators homogeneously entrapped in newly formed hemostatic plugs in the course of thrombolytic therapy. Because the premature dissolution of fibrin clots is probably responsible for the bleeding side-effects of thrombolytics, the similarity of t-PA and reteplase in this assay may explain the identical rate of hemorrhagic complications with these two activators [13].

In conclusion, our report provides evidence for the presence of free fatty acids in thrombi, which optimize the fibrin matrix as a template for plasminogen activation by t-PA and thus accelerate fibrinolysis with activators that approach the clot from the fluid phase. Fibrin also partially protects plasmin and t-PA against inhibition by oleic acid. In addition, a variant of t-PA (reteplase), which is a less efficient plasminogen activator in a pure fibrin environment, has an advantage over the wild-type molecule in the presence of fatty acids. Analogous evaluation (using assay formats that approach the complex composition of thrombi) may explain why there is no obvious correlation between fibrin binding and the *in vivo* fibrinolytic efficiency of recombinant plasminogen activators. Finally, the variability in the free fatty acid content of thrombi may contribute to the variable therapeutic outcome of thrombolysis in different patients.
